# Evolution of Spinal Cord Transection of Rhesus Monkey Implanted with Polymer Synthesized by Plasma Evaluated by Diffusion Tensor Imaging

**DOI:** 10.3390/polym14050962

**Published:** 2022-02-28

**Authors:** Axayacatl Morales-Guadarrama, Hermelinda Salgado-Ceballos, Israel Grijalva, Juan Morales-Corona, Braulio Hernández-Godínez, Alejandra Ibáñez-Contreras, Camilo Ríos, Araceli Diaz-Ruiz, Guillermo Jesus Cruz, María Guadalupe Olayo, Stephanie Sánchez-Torres, Rodrigo Mondragón-Lozano, Laura Alvarez-Mejia, Omar Fabela-Sánchez, Roberto Olayo

**Affiliations:** 1 Centro Nacional de Investigación en Imagenología e Instrumentación Médica, Universidad Autónoma Metropolitana Iztapalapa, CDMX, Mexico City 09340, Mexico; amorales@ci3m.mx; 2 Departamento de Ingeniería Eléctrica, Universidad Autónoma Metropolitana Iztapalapa, CDMX, Mexico City 09340, Mexico; ibqfabela@hotmail.com; 3 Departamento de Física, Instituto Nacional de Investigaciones Nucleares, Axapusco 52750, Mexico; gogol1000@hotmail.com (G.J.C.); guadalupe.olayo@hotmail.com (M.G.O.); 4 Instituto Mexicano del Seguro Social, Unidad de Investigación Médica en Enfermedades Neurológicas, Hospital de Especialidades Centro Médico Nacional Siglo XXI, CDMX, Mexico City 06720, Mexico; melisalce@yahoo.com (H.S.-C.); igrijalvao@yahoo.com (I.G.); phanie85@yahoo.com.mx (S.S.-T.); lau_alvarezmejia@yahoo.com.mx (L.A.-M.); 5 Centro de Investigación del Proyecto CAMINA A.C., CDMX, Mexico City 14050, Mexico; ruy.lozano@gmail.com; 6 Departamento de Física, Universidad Autónoma Metropolitana Iztapalapa, CDMX, Mexico City 09340, Mexico; jmor@xanum.uam.mx; 7 Investigación Biomédica Aplicada S.A.S. de C.V., CDMX, Mexico City 14240, Mexico; rhpithecus@yahoo.com.mx (B.H.-G.); ibanez.alejandra@hotmail.com (A.I.-C.); 8 Departamento de Neuroquímica, Instituto Nacional de Neurología y Neurocirugía Manuel Velasco Suárez S.S.A., CDMX, Mexico City 14269, Mexico; crios@correo.xoc.uam.mx (C.R.); aradiazruiz@hotmail.com (A.D.-R.); 9 Catedrático CONACyT-Instituto Mexicano del Seguro Social, Unidad de Investigación Médica en Enfermedades Neurológicas, Hospital de Especialidades, Centro Médico Nacional Siglo XXI, CDMX, Mexico City 06720, Mexico; 10 Departamento de Química Macromoléculas y Nanomateriales, Centro de Investigación en Química Aplicada, Saltillo 25294, Mexico

**Keywords:** plasma polymerization, spinal cord injury, diffusion tensor imaging, rhesus monkey

## Abstract

In spinal cord injury (SCI) there is damage to the nervous tissue, due to the initial damage and pathophysiological processes that are triggered subsequently. There is no effective therapeutic strategy for motor functional recovery derived from the injury. Several studies have demonstrated neurons growth in cell cultures on polymers synthesized by plasma derived from pyrrole, and the increased recovery of motor function in rats by implanting the polymer in acute states of the SCI in contusion and transection models. In the process of transferring these advances towards humans it is recommended to test in mayor species, such as nonhuman primates, prioritizing the use of non-invasive techniques to evaluate the injury progression with the applied treatments. This work shows the ability of diffusion tensor imaging (DTI) to evaluate the evolution of the SCI in nonhuman primates through the fraction of anisotropy (FA) analysis and the diffusion tensor tractography (DTT) calculus. The injury progression was analysed up to 3 months after the injury day by FA and DTT. The FA recovery and the DTT re-stabilization were observed in the experimental implanted subject with the polymer, in contrast with the non-implanted subject. The parameters derived from DTI are concordant with the histology and the motor functional behaviour.

## 1. Introduction

SCI has a great medical and socioeconomic impact, due to a severe neurological disability [1,2,3] and, so far it doesn’t exist an effective treatment to recovery after the injury. The pathophysiological mechanisms triggered after SCI are complex [2], small animal studies have contributed greatly to a better understanding of these mechanisms. However, it has not been possible to translate those findings effectively to improve treatments for human SCI. Therefore, to facilitate the translation of advances made in the laboratory to the clinic it is recommended the use of mayor species [4,5,6], including methods which allow the information collected per animal to be maximized in order to reduce the use of animals [7], prioritizing the use of non-invasive techniques to evaluate the injury progression with the applied treatments, according to organizations such as “The National Centre for the Replacement, Refinement & Reduction of Animals in research” (NC3Rs) in the UK’s that practice the principles of replacement, reduction and refinement (3Rs).

Polymers such as collagen/silk fibroin, polyethylene glycol, poly-*β*-hydroxybutyrate, chitosan tube, poly(ε-caprolactone), poly(lactic-co-glycolic acid), and polymers synthesized by plasma derived from pyrrole (PPPyI) have been proposed with encouraging results in the treatment of SCI [8,9,10,11,12,13,14,15,16], it has been described that polymers implanted after a SCI have beneficial effects such as: promoting functional recovery, axonal remyelination, preservation of nervous tissue adjacent to the epicentre of the injury, decrease in the number of reactive astrocytes, among others; although commonly the aforementioned effects do not occur together, in addition to not being attributed only to the polymer, but have been associated with the combination with drugs, cells or other agents [8,9,10,11,12,13]. While PPPyI has been reported to have neuroprotective and neuroregenerative effects *per se*. In addition, it was shown that animals with SCI transection (SCIT) implanted with PPPY presented a lower inflammatory response, better integration with the nervous tissue, and greater functional recovery compared to animals administered PPy synthesized by chemical or electrochemical methods. This can be attributed to the plasma synthesis of the polymer, with which polymers with physical-chemical characteristics different are obtained [17]. Additionally, it has been shown that combining rehabilitation with the PPPyI implant promotes the expression of βIII-tubulin (molecule related to nerve plasticity), reduces glial scar formation, favours the preservation of nerve tissue, nerve fibres cross the injured site and recovery of motor function is observed [16]. PPPyI implanted in adult rats with SCI by both, transection and contusion models, favour protection of nervous tissue adjacent to the lesion and the functional recovery of animals, significantly [14,15,16].

Magnetic Resonance Imaging (MRI) has been used as a non-invasive tool in order to study the SCI in vivo. With conventional MRI, for instance T2-weighted (T2W), it is possible to obtain morphometric information of the injured area, the affected tissue and the conformation of cysts and scar areas [18,19,20]. However, to evaluate the severity of SCI, more sensitive methods are needed to reveal changes in the neurological structure, as DTI that has been developed and used as an accurate, non-invasive evaluation approach in SCI [19,21,22]. DTI is an MRI technique with the possibility to determine the direction of water diffusion in biological tissues. In white matter, water diffusion is highly directional through axons [23], DTI can be used to assess the microstructure of white matter by FA, which reflects the anisotropy of the diffusion [24]. DTT refers to the estimation of axonal connectivity according to local diffusion properties. In its simplest form, DTT follows the main axis of the nerve fibres and their propagation from one area to another anatomically connected [25,26,27].

In this work experimental T9 SCIT was performed, and the evolution through time by DTI of SCIT in nonhuman primates (NHP) at the epicentre and around the injury was studied.

## 2. Materials and Methods

### 2.1. Synthesis of Polypyrrole Iodine by Plasma

The monomer used in the polymerizations was pyrrole (99%, Sigma-Aldrich, St. Louis, MO, USA), the dopant was iodine (99.8%, Sigma-Aldrich, St. Louis, MO, USA). Polypyrrole Iodine was synthesized by the plasma polymerization method. The film was kept in the reactor for 24 h. In an iodine atmosphere to neutralize the last free radicals and increase the amount of iodine in the material. The pyrrole and iodine used in the synthesis and the solvents applied to remove the polymers were used without further purification. Once the PPPyI films were synthesized, acetone was applied to separate the films from the substrates and dissolve the remaining oligomers. FT-IR, XPS, TGA and morphological analyses were performed (its characterization has already been reported in previous studies [14,28,29]). When removing the polymer from the reactor, flakes of around 1mm thickness are obtained, films were pulverized in an agate mortar to obtain a fine brown powder, which was compressed at 9 tons for 10 min to form a thin tablet. Electrical conductivity was calculated from resistance measured directly from the tablet [14,28].

### 2.2. Polymer Characterization

The analysis FT-IR of the polymer was obtained with a Perkin-Elmer 2000 Infrared Spectrophotometer (Perkin Elmer-DuraSampIIR II, Waltham, MA, USA) collected directly from the films through 32 scans. PPPyI films were analyzed by X-ray photoelectron spectroscopy (XPS) using an X-ray monochromator from an Al kα (1486.6 eV) source (Thermo hermo Scientific, Waltham, MA, USA).

Resistance of the tablet was measured perpendicular to the polymer surface using a two-probe device, the sample placed between two copper electrodes in a capacitive array. The resistance of the polymer was measured with a multimeter.

### 2.3. Animal Grouping

This study was approved by the National Commission of Scientific Investigation of Mexican Social Security Institute, The Committee for the Care and Use of Laboratory Animals of Proyecto CAMINA A.C. research centre, and Bioethics Committee of National Centre of Investigation in Medical Instrumentation and Imaging. The NHP were treated humanely according with “National Institutes of Health Guide for the Care and Use of Laboratory Animals”.

Two female NHP (Macaca Mulatta) from CAMINA A.C. were used. They were selected according to their general healthy aspect, mobility, vital sings, and blood chemistry. The NHPs underwent the SCI, one of them was implanted with the polymer (RHI) and the other one was only injured (RHC). A month before starting the experimental procedures, NHP were moved from their troop to a cage. They were maintained *ad libitum* with *pellets* Purina Monkey Diet 5045^®^ (PMI Nutrition International, St. Louis, MO, USA) and water. To safely handle NHPs, *Pole-and-Collar* systems (Primate Products Inc., Immokalee, FL, USA, EE.UU.) were used.

### 2.4. Experimental Treatment

NHPs were induced with tiletamine zolazepam (Zoletil, Virbac S.A., Carros, France) intramuscularly (4 mg/Kg). Isoflurane anaesthesia (Rhodia Organique Fine Ltd., CDMX, México) was kept at 1.5% providing oxygen mixed with environmental air though an endotracheal tube (approximately at 25 mL/s). Physiological parameters were monitored: rectal temperature, oxygen saturation and ECG, during the surgery. The ventilator was maintained at 15 respirations per minute. For the SCI, the isoflurane concentration increased to 2.5%. Afterwards the NHPs were subjected to a sagittal incision on the skin and to a dissection in the paravertebral muscles to carefully remove one lamina. In order to observe the laminar process of this vertebra, the ninth spinous process was extirpated. Then a laminectomy was carried out, being extended until the facet process. So far, the meninges were kept intact. Once the laminectomy was finished a longitudinal incision was carried out in the meninges. After that, a complete transversal cut in the spinal cord was carried out and the PPPyI implant was introduced into the lesion site in the RHI subject. Afterwards the meninges were sutured with stitches and the surgical incision was sutured in two planes. Immediately after the injury, MRI studies were performed. Ciprofloxacin Lactate (Bayer de México S.A. de C.V., CDMX, México) was administrated intravenously every 12 h the first day after the injury and then a daily intramuscular dose of 15 mg/kg for 6 days, as prophylactic to prevent infections. For Analgesia, a daily dose for two weeks of 100 mg/kg of Acetaminophen (Cilag, México) was administrated orally.

### 2.5. MRI and DTI Scan

MRI studies were performed with a 3.0 T whole-body MRI clinical scanner (Achieva, Philips Medical Systems, Eindhoven, Netherlands) with 4-channel SENSE coil.

T1-Weigthed was obtained: PROSET-CLEAR sequence; TE/TR 6.9/10.9 ms; FOV 196 × 132; matrix 300 × 200; slice thickness 1mm.

T2-Weigthed: VISTA-CLEAR sequence; TE/TR = 115/2500 ms; FOV 196 × 132; matrix 300 × 200; slice thickness 1mm.

DTI was acquired with the following parameters: DTI-high_iso sequence; TE/TR 70/5934 ms; b = 800; 32 directions; FOV 128 × 128; matrix 256 × 256, slice thickness 2 mm.

The studies were carried out: before SCI, the day of the SCI and the 1st, 2nd, and 3rd month after SCI. The data were then further analysed using DSI Studio software (7 January 2021 build, Fang-Cheng, Pittsburgh, PA, USA), The diffusion tensor was calculated. A deterministic fibre tracking algorithm was used [30], the regions of interest were placed at the epicentre (ECn), the rostral direction (Rn), and the caudal direction (Cn) of the injury. The seeding region was placed around the epicentre of injury. The anisotropy threshold was 0.225. The angular threshold was 30 degrees.

### 2.6. Obtaining Tissue

Three months after the SCIT, subjects were anesthetized followed by an intraperitoneal administration of 0.8 mL of heparin, a wide thoracotomy was performed, the ascending aorta was cannulated, and 1000 mL of cool physiological saline solution followed by 2000 mL of 4% paraformaldehyde in phosphate buffer were perfused through the heart. The spinal cord was obtained. The samples were embedded in paraffin. Serial longitudinal sections of 10 μm thickness was cut and stained for histological analysis.

## 3. Results

### 3.1. FT-IR Spectcroscopy

**Figure 1:**
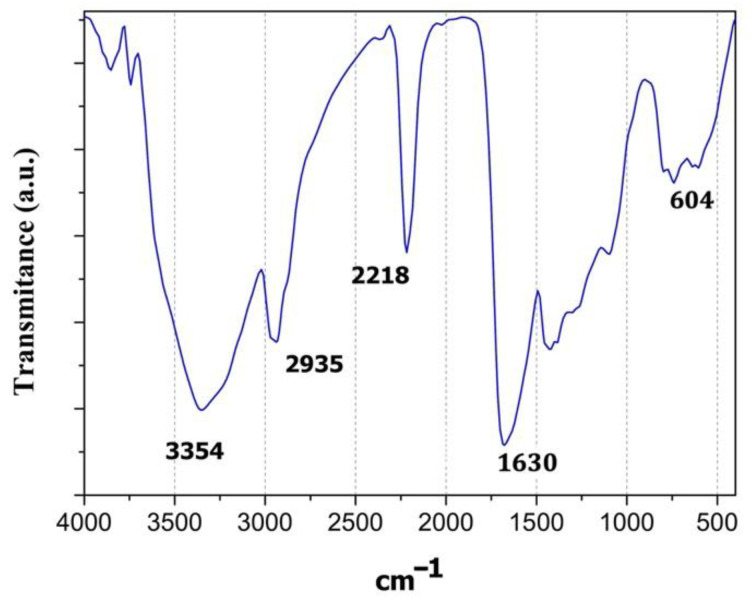
Infrared spectrum (FT-IR) of iodine-doped polymer of pyrrole synthesized by plasma.

The infrared spectra of PPPyI are shown in Figure 1. Three broad absorption bands are primarily identified and emphasized. Absorption region located in the interval 400–800 cm^−1^, shows the substitutions in the pyrrole rings, associated with the crosslinking between the chains, the partial branching, and the growth of the polymer. The C–I groups originating from iodine doping during polymerization can be identified in the peak centred at 604 cm^−1^. Wide absorption located in the region between 1600 and 1800 cm^−1^, is due to the C–N, C=C and C=0 groups, the peak at 1630 belongs to the amine group. The vibration at 2218 cm^−1^ corresponds to nitrile groups, C≡N, this vibration suggests high dehydrogenation and breakage of some monomeric rings. The aliphatic C–H groups can be assigned to absorption centred at 2935 cm^−1^ and suggest the ring fragmentation, as result of plasma synthesis high-energy collisions. In the region between 3000 and 3800 cm^−1^ may be associated to N–H and O–H groups, the most significant absorption is centred at 3354 cm^−1^, which corresponds to the pyrrole bonds N–H.

### 3.2. Elemental Analysis by XPS

**Figure 2:**
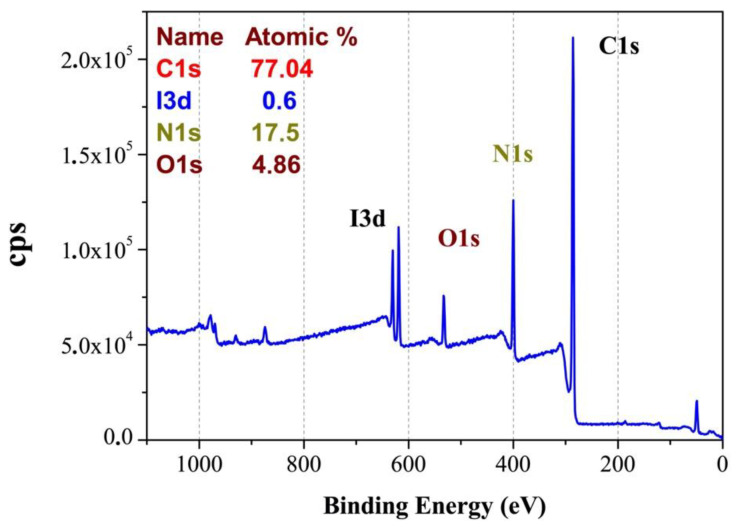
XPS survey spectrum for PPPy/I. The peaks correspond to configurations of carbon (C1s), nitrogen (N1s), oxygen (O1s), and iodine (I3d).

Atomic percentage analysis of PPY/I indicated C 77.04%, O 4.86%, N 17.5% and I 0.6%; as is showed in Figure 2. Carbon and Nitrogen are part of the pyrrole structure, while the Iodine has a low participation due to its integration as a dopant during the synthesis. On the other hand, Oxygen can be a consequence of the neutralization of the last free radicals when the reactor is opened and exposed to the atmospheric interaction.

### 3.3. Electric Conductivity

The electrical resistance of the PPy/I tablet was 1.4 MOhm at 30% relative humidity and the associated conductivity was 2.2 × 10^−10^ S/cm, these results are consistent with those reported [14]. Some PPPYI, follow an ohmic behaviour, which increases several orders of magnitude when the relative humidity is >60% [28]. This behaviour is desired since the material is implanted in a medium surrounded by body fluids.

### 3.4. Implant Evolution

**Figure 3:**
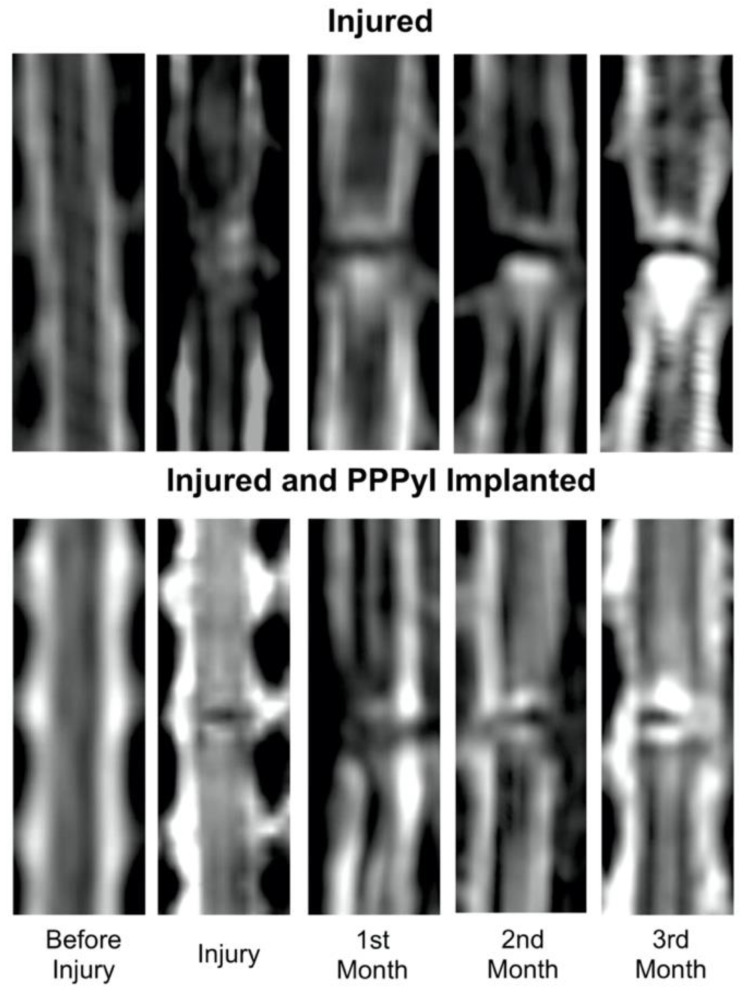
T2-weighted magnetic resonance imaging study of Rhesus injured and injured + implanted. T2W sagittal reconstruction images show transection site from before injured up to 3 months after injury.

T2W show the transection site and its changes around the injury (Figure 3). In the rhesus injured (RHC), cysts can be observed in rostral direction from the injury epicentre, increasing size over time. The rhesus injured and implanted (RHI) T2W images show a hyper intense region around the implant, which does not extend.

**Figure 4:**
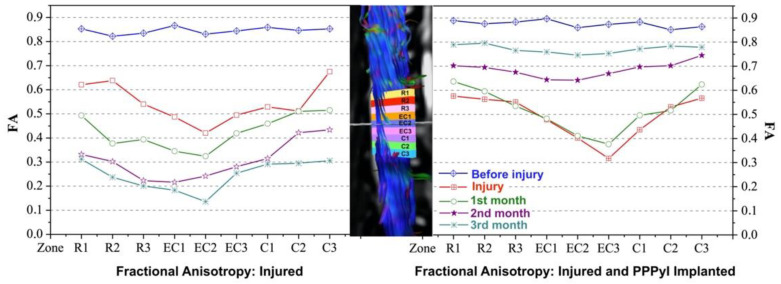
Fractional anisotropy of injured area. The FA was measured in 9 different regions of the transection area, shown in the middle of the figure, where ECn corresponds to the epicentre of injury, Rn is the rostral, and Cn is the caudal region. The time evolution graphs of the values of FA of the RHC (**left**) and RHI (**right**) are presented.

FA was measured in different areas around the injury site (Figure 4). The values of FA in the different regions measured before the lesion are larger than 0.8 mm for both, RHC and RHI. This value shows a unidirectional diffusion with high FA. After the injury the evolution in time of RHC, and RHI are different. While in RHC a tendency to FA decreasing in the injury area was observed from the 2nd month, in RHI there is an increasing FA. FA for the RHC before the injury was 0.83 ± 0.02. The injury day, FA decreased to 0.42 ± 0.02 at the injury epicentre, and as the injury was progressing FA drops. One month after the injury FA was 0.32 ± 0.02, two months after the injury was 0.24 ± 0.02 and 3 months after the injury was 0.14 ± 0.02. In RHI, FA was 0.86 ± 0.02 before the injury, the day of injury and implant FA decreased to 0.40 ± 0.02 at the injury epicentre, one month after that, FA was 0.41 ± 0.02, after two months it was 0.64 ± 0.02, and three months post injury was 0.74 ± 0.02. At the end of the study, the FA in RHC decreased 86% while for RHI it decreased 15%.

**Figure 5:**
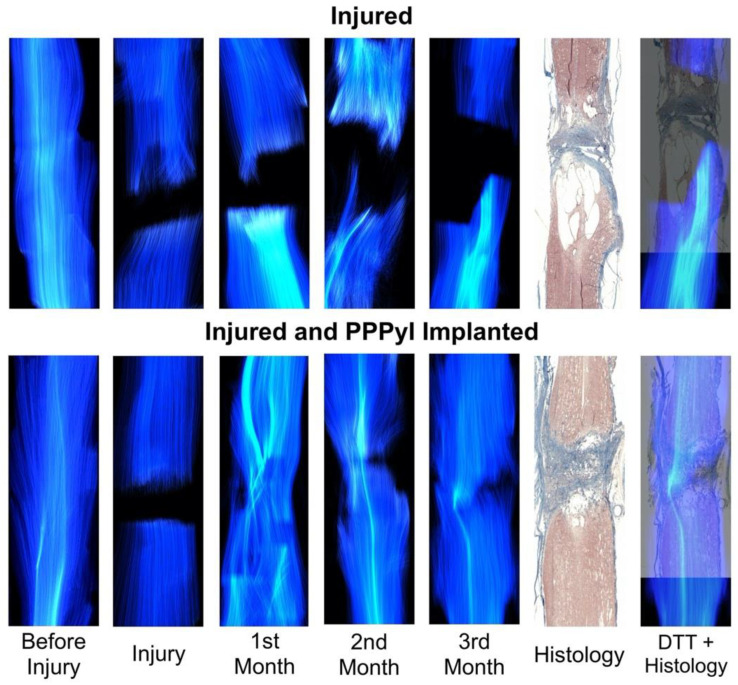
Diffusion Tensor Tractography of Spinal Cord Injury through time. The evolution shown is from before the injury to 3 months later. In the injured Rhesus, shown above, the anisotropy decreases with time, showing an increasing discontinuity in the fibre- tracking to both sides of the lesion. In the injured and implanted Rhesus, shown below, tracts are identified through the injured area, showing a gradual decrease in discontinuity and a progressive recovery of the tractography over time.

DTT of the SCI region allows visualizing changes in the white matter through time (Figure 5). The before injury column shows a well-organized fibre-tracking in both, RHC and RHI. For the first image after the injury, discontinuity in the projection of the tracks is observed in both subjects. In the RHC, fibre-tracking integrity decreases over time (from 1 to 3 months) in rostral and caudal direction, extending to the ends of the analysed region. Restructuring of the fibre-tracing was observed in RHI, from the first month after the injury, showing irregularity and discontinuity in the fibre-tracing mainly in the epicentre of the injury, at the second month homogeneous lateralized fibres were observed at the second month the formation of homogeneous lateral fibres were observed, with tendency to integration, for the third month after the injury, the fibre-tracing is homogeneous in the lateral portion shown in the second month and the tendency to restructure on the opposite side is observed (see Supplementary Video).

Histology (Figure 5) show scar formation at the epicentre of the injury, as well as cyst formation in both the rostral and caudal directions. For RHC there is coalescence of cysts forming a large cyst, thus contributing to the loss of histoarchitecture, while for RHI there is no extension of the injury area, and integration of scar, PPPyI, and tissue is observed at the epicentre of the injury.

DTT and Histology overlap (Figure 5) also shows a composition of the DTT and histology, this combination has not total synchronization but allows to compare the morphology predicted by the two techniques, and they show congruency giving a clear idea of the difference in of the evolution of the injury in the subjects.

**Figure 6:**
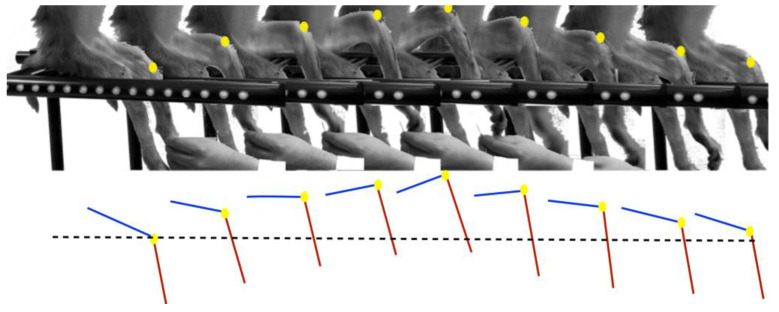
Movement recovery in implanted NHP. The one limb movement trajectory of the RHI is observed 3 months after the polymer implantation. Below, the movement kinetics representation of the relation Hip-Knee-Ankle, the dotted line shows the knee initial position.

The recovery of sensitivity and movement in the lower extremities, was observed only in RHI since RHC did not show any motor recovery and presented lacerations, sores, muscle atrophy, and therefore no movement is discussed; evidence is omitted due to the shocking and crude nature of the injuries. In the 2nd month, RHI subject had slight movements of the lower joints, while in the third month, complete flexures were observed, particularly of the relation Hip-Knee-Ankle (see Supplementary Video), Figure 6 shows the movement trajectory of a lower extremity evaluated 3 months after injury, and their kinetics representation.

## 4. Discussion

In this work a SCIT was carried out in non-human primates, one with implanted of PPPyI (RHI) and other just with the injury (RHC), the effect of the PPPyI in RHI is in accordance with the previous work in murines, in which, the use of PPPy in different models of SCI has promoted mechanisms of neuroprotection, contributing in the stimulation of motor plasticity mechanisms and in the histoarchitecture conservation, increasing the motor functional recovery of the experimental subjects [14,15,16]; DTI showed capacity to evaluate the evolution in vivo of the SCIT in NHP emphasizing the difference between the RHI and RHC.

FA is a sensitive marker for SCI and is strongly related to the severity of the lesion [21]. FA can be an indicator of axonal structure damage with the possibility to quantify the severity and extension of SCI. The decrease in FA reflects axonal loss and nervous degeneration. FA decreased the injury day for the subjects in the epicentre of the injury. Subsequently, FA in RHC kept decreasing in the epicentre, and in all the evaluated regions showing a larger damage, FA value was 0.14 ± 0.02 in the epicentre three months after the lesion, agreeing with the tendency in the decrease of FA reported in murine [21,31] and canine [32] with SCI. In RHI a gradual recovery of FA after the second month is observed and the fraction increased up to 0.74 ± 0.02 at the epicentre and showed a tendency to recover the value of both, caudal and cephalic, suggesting a re-stabilization of the microenvironment in the lesion zone [31,33].

DTT can differentiate the interrupted nerve fibres from intact regions and can be used as a qualitative indicator of SCI to represent nerve fibres and to observe the spinal cord evolution after an injury, the fibres tracking is directly related to the change in FA, since as tracts are damaged the anisotropy decreases [21,31,32,33,34]. In both subjects, the DTT projection of the initial spinal cord interruption on the day of the injury was observed. The decrease in anisotropy can be observed in the RHC, where gradually there was an increase in the separation between each extreme of the spinal cord, for the 3rd month after the injury, the space is extensive which is consistent with the space generated by the cysts, according to what is shown in the histology, the DTT + Histology overlap, demonstrates how it is possible to visualize the extent of the lesion in vivo in RHC by means of DTT. In RHI, DTT shows a tendency to recover the track continuity in the injury area because of the FA recovery, showing also local changes in the directionality through time, for the first month, the DTT projection shows a large number of incomplete tracts in the epicentre of the injury, this is attributed to the change in the anisotropy of the region due to the presence of the PPPyI and its interaction with the tissue. Subsequently, greater homogeneity is observed in the area, and the existence of calculated tracts due to the increase in FA in the area, this being coincident with the reappearance in the 2nd month of slight movements in the subject of the lower joints, for the 3rd month, the DTT projection shows a greater number of tracts calculated in the epicentre of the injury as well as changes in the local directionality, analogously in the histology, interaction between tissue and scar is observed in the epicentre of the injury, the DTT + Histology overlap suggests the coincidence of this interaction and the DTT projection, as well as the recovery at the 3rd month after the injury of complete flexures and sensitivity.

The analysis of DTI/DTT has the ability to monitor in vivo the state of the tissue surrounding the epicentre of injury, as well as quantifying the anisotropy of the same region in non-human primates after SCI. In humans, the MRI studies commonly used in SCI are qualitative, limited only to determining the morphology of the Injury, therefore the use of DTI/DTT in the clinic would be an important tool to evaluate and follow up SCI in humans.

In conclusion, diffusion tensor imaging is sensitive to evaluate spinal cord injury, allows monitoring of in vivo injury in nonhuman primates, serving as a tool to evaluate the progression of the injury through time.

## Data Availability

The data presented in this study are available on request from the corresponding author.

## Supplementary Materials

The following supporting information can be downloaded at: https://www.mdpi.com/article/10.3390/polym14050962/s1.
